# Resident Practice Audit in Gastroenterology (RPAGE): an innovative approach to trainee evaluation and professional development in medicine

**Published:** 2019-07-24

**Authors:** Sandra Monteiro, Ted Xenodemetropoulos

**Affiliations:** 1 McMaster University, Ontario, Canada

## Abstract

**Background:**

The Resident Practice Audit in Gastroenterology (RPAGE) captures assessments of knowledge, professionalism, and technical skills, in real time. This brief report describes this innovative instrument and aspects of its utility.

**Methods:**

Assessment data on colonoscopy, endoscopy, and sigmoidoscopy procedures in 2016 were submitted to a repeated measures ANOVA with six within subjects’ assessments and one between subjects’ factor of year of specialization to evaluate construct validity. The validity hypothesis tested was that more experienced residents would be rated higher than less experienced residents. Reliability was assessed using Cronbach’s alpha.

**Results:**

The proportion of completed assessments was relatively low (9 to 22%). Overall reliability was high (α >0.8). There was evidence of validity as global ratings indicated higher competence for senior residents at colonoscopy (1.6) and upper endoscopy (1.4) than for more junior residents (1.9 and 2.1 respectively). These differences were significant for both colonoscopy, (F (1, 282) = 14.8, p <0.001) and endoscopy, F (1, 136) = 56.9, p <0.001.

**Conclusion:**

These findings suggest RPAGE is an acceptable electronic log of practice data, but may not be acceptable for workplace based assessment. A key next step will be to evaluate how information collected through RPAGE can help inform resident competency committees.

## Introduction

The Competence by Design (CBD) initiative of the Royal College of Physicians and Surgeons of Canada^1,2^ is a phased plan to develop training models with an enhanced focus on trainee accountability and demonstration of performance. The success of this plan requires objective, validated mechanisms for assessing continuous performance improvement and professional development. While, several authors challenge the conceptual basis for CBD,^3-5^ and others note challenges with assessing technical skills,^6-8^ there is little resistance to improving the capture of assessment data.

Logistically, paper-based assessment forms are difficult to customize by procedure resulting in either cumbersome or overly simplistic assessment.^9^ Additionally, challenges exist when integrating data across multiple paper forms limiting translational benefits to both trainees and evaluators. Finally, paper-based forms limit the recording and interpretation of real-time feedback.^10,11^ Electronic data capture offers an effective solution to these problems, but practical challenges remain.

The resident practice audit in gastroenterology (RPAGE) instrument is a customized electronic platform designed for real-time data capture of competence assessments and procedure logs for medical residents specializing in gastroenterology (GI) in Canada. The RPAGE instrument affords an opportunity to evaluate some of the assumptions inherent in the CBD framework. Specifically, we were interested in whether creating an improved system of data capture would ensure reliable and valid assessments or facilitate the use of learning analytics.^2,12^

### Background of the RPAGE concept

The RPAGE program was first introduced to Canadian Gastroenterology Residency Training programs at the annual McMaster University First Year Residents Endoscopy Course held in July of 2011. Group orientations to the RPAGE instrument were conducted at McMaster University. Participants were oriented to the use and functionality of RPAGE as well as the objectives of the present study. Participants included trainees in the Adult Gastroenterology Training Program , attending faculty members within the Division of Gastroenterology and all endoscopy unit nurses.

Previous experience with an online colonoscopy assessment instrument developed for the Canadian Association of Gastroenterology's Quality Program in Endoscopy had demonstrated positive uptake and usage by endoscopists.^13,14^ New formats integrating competence assessment extending beyond technical skills (including professionalism, teamwork and time management) were suggested as a means for providing a multidimensional evaluation.^14,15^ This paper describes the first step to evaluate RPAGE for use in real-time. Our goal was to determine whether RPAGE was being used as an assessment tool, and whether the assessment data being recorded were informative. This study explores the first full year of data using the RPAGE system at McMaster University and reports on its acceptability, validity, and reliability. The results offer some answers and open additional questions that may continue to challenge the implementation of CBD.

## Methods

### Instrument design and mechanism of data capture

A demonstration of the RPAGE upper GI endoscopy, flexible sigmoidoscopy and colonoscopy evaluation instruments can be accessed at http://www.cag-rpage.org (username: demo; password: resident). Separate, comprehensive RPAGE modules for diagnostic and therapeutic upper GI endoscopy, flexible sigmoidoscopy and colonoscopy were designed to collect relevant demographic, pre- procedural and procedure-specific information, adopted from current endoscopic credentialing and quality guidelines from the Canadian Association of Gastroenterology. The architecture of the RPAGE instruments follows the model for the Canadian Association of Gastroenterology Quality Program for Endoscopy (which was granted an Innovation Award by the Royal College of Physicians and Surgeons of Canada).

Although each assessment can be customized to the procedure and the needs of both the trainee and patient case, all residents/procedures can be evaluated for professionalism, knowledge of the procedure, procedure completion, technical skill, interpretation and management, patient safety and a global assessment using a 4-point Likert scale based on independence and competence. The anchor labels are: 1) Highly skilled advanced performance of all tasks; 2) Competent for independent performance of all tasks without the need for any guidance; 3) Achieves most of the tasks independently, with minimal verbal and/or manual guidance; and 4) Achieves some of the tasks but requires significant verbal and/or hands-on guidance. The instruments can be accessed via desktop and mobile computer platforms. We assigned touchscreen tablets (GalaxyTab 7.0 and 10.0, Samsung Inc.) supporting wireless connectivity for data transfer to each Gastroenterology trainee throughout the period of study.

### Evaluation

For the purpose of this study, we restricted our analyses to describing available measures of acceptability, reliability and validity. Exploration of additional components of utility, such as generalizability and cost are ongoing. This study includes data from January 2016 to December 2016. To evaluate reliability, we calculated Cronbach’s alpha, as an indicator of whether the scores could discriminate between individuals of different clinical competence levels. To evaluate acceptability, we hypothesized that real-time functionality with simplified data entry would result in a large proportion of all logged procedures containing usable assessment data for the core competencies within each module: professionalism, interpretation of data, patient safety, knowledge, independence, and technical skill. To evaluate validity, we used a hypothesis testing approach. It may be worth noting that in GI, “junior” residents are in their fourth or fifth post-graduate year when they start their sub- specialty training and typically take two to three years to achieve independent practice. We hypothesized that trainees in their first year of specialization would receive lower scores, based on group averages, than trainees in their second year of specialization. We hoped to capture assessments of trainees in all three years of training.

In RPAGE, competence scores closer to 1 (Highly skilled advanced performance of all tasks) are an indicator of higher competence, while scores closer to 5 indicate a lower competence level. Case difficulty was rated on a 5-point scale from extremely easy (1) to very difficult (5). Global assessments were submitted to a univariate ANOVA with one between- subjects factor of training level. Competency scores were submitted to a repeated measures ANOVA with one between-subjects factor of year of specialization and one within-subjects factor of competency at 6 levels: professionalism, interpretation of data, patient safety, knowledge, independence, and technical skill. Finally, competency scores were evaluated using Cronbach’s alpha as a preliminary evaluation of the reliability of the assessment process. The Hamilton Integrated Research Ethics Board determined, this project was exempt from formal REB review, having been designed as a quality improvement and education program.

## Results

Data collection and analyses were completely devoid of any patient identifying information. In 2016, there were 19 active RPAGE access IDs for McMaster University medical residents specializing in GI. Of the 19 residents in this study, 10 residents progressed from *junior* to *senior* within the program in 2016, and nine remained at the junior level. The average scores for the six competencies are reported in Table 1. Overall, the group received higher scores for professionalism than technical skill, which may be seen as appropriate for trainees. Further investigation into assessment trends can offer further interpretation.

**Table 1:** Mean competency ratings in Professionalism, Interpretation of data, Patient safety, Knowledge, Independence, and Technical skill for Colonoscopy and Endoscopy

Procedure	Professionalism	Interpretation of Data	Patient Safety	Knowledge	Independence	Technical Skill
**Colonoscopy**	1.30	1.94	1.61	1.92	2.25	2.25
**Endoscopy**	1.14	2.07	1.69	2.03	2.14	2.15

Mean competency ratings for residents in year 1 and 2 that logged usable assessments for Colonoscopy and Endoscopy. **Lower** scores indicate **increased** competence. The anchor labels for the scoring are: 1) Highly skilled advanced performance of all tasks; 2) Competent for independent performance of all tasks without the need for any guidance; 3) Achieves most of the tasks independently, with minimal verbal and/or manual guidance; and 4) Achieves some of the tasks but requires significant verbal and/or hands-on guidance.

### Acceptability

Although RPAGE was designed for any health professional to use (as an assessor), all ratings were provided by GI faculty. In total, McMaster University GI residents logged 2636 patients/procedures in 2016. However, we were limited in our ability to fully evaluate the usability and acceptability of RPAGE as it was not possible for us to determine how many procedures were performed that year in total. For the purpose of this study, this large number of logged procedures contributed positively to our evaluation of RPAGE as an audit tool, as these were only recorded at one site in Hamilton health care center. But the data revealed a different perspective regarding acceptability for assessment. While 1263 colonoscopy procedures were logged, there were only 284 (22%) assessments of competence. Similarly, 1281 upper endoscopies were logged with competence assessments reported for 138 (11%) procedures. Finally, there were 92 sigmoidoscopies logged in RPAGE, with competence assessed for eight (9%) procedures (performed by four different senior residents). Notably, not all residents logged procedures for both colonoscopy and endoscopy in both years. For example, some residents were assessed on colonoscopies in year one and two, but only in year one for endoscopies. Since we did not have data from all residents from year one and two, we did not formally analyze individual progress across training year; there simply was not enough data for that analysis to be conducted.

### Reliability

The scores demonstrated strong reliability as Cronbach’s alpha was > 0.8 for both the Colonoscopy and the Upper Endoscopy modules; this indicates that the scores were able to discriminate between individuals, who may vary in levels of competence.^16^ As Endoscopy was logged only eight times, we did not evaluate reliability.

### Validity

The RPAGE did capture some trends on an individual level. For the 10 residents who were in the RPAGE as junior and senior trainees, eight improved in performance over time. We noted two residents showed a decrease in scores on a global assessment of colonoscopy procedures. As this was an anonymized study, we could not follow up on those residents and while it was not appropriate to evaluate this pattern statistically, it is possible that such indicators can be used by program directors to identify medical residents in need of remediation. Scores for sigmoidoscopy were not analyzed as there were only eight assessments for four residents in year 2.

Average ratings were higher for senior residents on colonoscopy (1.72) and endoscopy procedures (1.49) than more junior residents (2.03 and 2.24 respectively). These differences were modest but significant for both colonoscopy, (F (1, 282) = 11.79, p <0.001) and endoscopy, F (1, 136) = 71.07, p <0.001. Conversely, case difficulty was rated as higher for residents in their first year of specialization in endoscopy than for residents in their second year (3.17 vs 2.72), F (1,136) = 10.44, p < 0.001 and colonoscopy (3.44 vs 3.07). F (1,282) = 7.91, p<0.01. This observation may also be seen as a sign of validity as determinations of case difficulty are certainly context specific; a more experienced physician will proceed as if the case is easy while for a novice the same case will be difficult. Unfortunately, we do not know how the examiners were evaluating case difficulty and this may be an issue to follow up on through examiner training.

## Discussion

This study set out to evaluate RPAGE as an assessment tool and to determine if the scores were reliable and valid. While reliability and aspects of validity were established, the actual story is more complex.^12^ The discrepancy between logged and usable records suggests that both faculty and trainees were primarily using the audit tool to count procedures, rather than to provide detailed assessment and feedback data. From one perspective, uptake may be considered successful as residents could track their experience with different procedures and patient demographics. From another perspective, RPAGE is not yet an acceptable workplace-based assessment tool. As programs across Canada embark on the path towards CBD, this study may offer some insights towards potential challenges and solutions.

The notable amount of missing data did come as a surprise to us but is not unusual.^16^ Despite the importance of competence assessment and the eventual nation-wide implementation of CBD, only about 16% of all procedures were assessed in any detail. It is possible that faculty self-regulated the need to evaluate performance, compared to ensuring that procedures were logged for future auditing purposes. That is, there may have been informal ratings of competence that were being made that pre-empted the decision to complete the assessment form in a formal manner.^13,14^ One thing that is certain, is that increased focus on competency by design and investment in a customized assessment instrument and local investment from the department chair and program director is not sufficient to ensure buy-in from faculty and residents. These results indicate that additional strategies are needed to invest in faculty development and ensure consistent adoption of assessment tools like RPAGE. Continued exploration of the data will be useful for evaluating the success of this audit, to determine which procedures are either not being performed by residents or are not being assessed. The pattern of assessments that are logged or not logged can itself be meaningful.^17^ Future work can help understand whether assessments were not formally recorded for specific reasons, such as time, pre-determined confidence in the trainee’s ability or the opposite - lack of confidence in the trainee’s ability and perceived need to be at their side for the entire procedure without attending to an assessment.

The assessment scores were determined to be reliable (α>0.8). The results also suggest validity was demonstrated, based on the hypothesis that senior residents would receive higher scores than junior residents. Unfortunately, there remains ambiguity around the faculty’s frame of reference.^18^ For example, we were unable to determine how case difficulty was evaluated in the context of subjective elements of faculty perspectives.^17^ It may be that faculty were simply noting that cases were generally more difficult for junior trainees, which is commensurate with the literature on expertise.^19^ Therefore, they may not have used a frame of reference that evaluated case difficulty objectively, but rather contextually. As case difficulty is often defined relative to experience level, this finding may be consistent with the validity hypothesis: residents that are more senior are seen as more competent and consequently cases appear easier for them. In order to better differentiate between case difficulty and competence, it may be necessary to include examiner training for these procedural skills that defines the frame of reference by routine or complex cases, rather than case difficulty.

There are certainly several limitations to this study. We were unable to track all patient procedures to determine if RPAGE was capturing everything. We were only able to report the total number within the system. Another major limitation is that RPAGE was not used consistently, thus limiting our ability to fully evaluate RPAGE as a formal assessment tool. The current dataset in RPAGE is not robust enough to track competence over time for all residents or procedures equally. Finally, we were unable to link the quantitative data with subjective reports from faculty or residents regarding their perceived utility of RPAGE. Hopefully, future work can examine how attitudes towards CBD assessment may impact acceptability and validity. Indeed, RPAGE is an *audit* and as such, we feel we have commented on the reliability, validity, and acceptability of the tool. It may take time and additional incentives before all stakeholders treat RPAGE as a true assessment tool as well. For now, RPAGE may facilitate the collection of competence assessments and procedure logs that can complement decisions around advancement.

### Conclusion

With a continued focus on real-time competency- based assessments, the need for tools like RPAGE will grow. With all specialty postgraduate medical training programs in Canada actively integrating competency- based medical assessments, audits of procedures completed will become integral to the decisions of evaluators and decisions about licensure. If certain procedures are not being logged due to lack of access (i.e., those kinds of patients are not being sent to a specific care center) training programs may need to consider the use of other curriculum-based interventions such as simulation or variable site rotations in order to help trainees acquire the requisite opportunities. Our study demonstrated that RPAGE was acceptable as an audit, and achieved minimal indicators of validity and reliability. However, it still needs work to improve usability and strengthen the potential as a true assessment tool.
